# Do people residing in low socioeconomic areas engage with and benefit from digital mental health services?

**DOI:** 10.1016/j.invent.2025.100865

**Published:** 2025-08-09

**Authors:** Lauren G. Staples, Blake F. Dear, Olav Nielssen, Nickolai Titov

**Affiliations:** MindSpot Clinic, Macquarie University, Sydney, Australia

**Keywords:** Digital mental health, E-mental health, Socioeconomic status, Treatment outcome, Service use, Barriers to care

## Abstract

**Background:**

People who are socioeconomically disadvantaged have higher rates of mental disorder and are more likely to face barriers to evidence-based psychological services. Barriers include the cost of treatment, limited availability of local services, and the burden of psychosocial difficulties. Psychological treatment delivered via the internet can overcome some of these barriers.

**Methods:**

This study was a retrospective analysis of data collected from 21,561 patients accessing online psychological assessment and treatment. Residential postcodes were used to assign patients to a socioeconomic group (low, mid, or high SES), based on the Index of Economic Resources published by the Australian Bureau of Statistics.

**Results:**

The low SES group comprised 34.1 % of the sample and the mid SES group comprised 35.7 %. A perceived lack of local or affordable mental health services was the main reason given for accessing online mental health assessment and treatment. There were small but significant demographic differences between groups at assessment, and baseline symptoms of depression and anxiety were slightly higher for the low and mid SES groups. Despite these differences, there were no group differences in treatment outcomes. All groups showed large symptom reductions on measures of depression (PHQ-9) and anxiety (GAD-7), with Cohen's d effect sizes between 1.36 and 1.47. Reliable deterioration rates were low, and satisfaction rates were high.

**Conclusion:**

This study shows that people residing in low socioeconomic areas engage with and benefit from digital mental health services. Results suggest that scalable digital psychological services can improve the equity of access to mental health care.

## Highlights

• Socioeconomic disadvantage is associated with higher rates of mental disorder
• Socioeconomic disadvantage can affect access to psychological services
• Digital psychology services can provide effective and acceptable support to patients across socioeconomic levels

## 1 Introduction

Equity of access to health care is a guiding principle of the Australian health system (Australian Commission on Safety and Quality in Health Care). However, socioeconomic status (SES), comprised of factors such as education, occupation, and financial security, is known to affect access to health care, including mental health care (Amos et al., 2023). Low SES is associated with an increased chance of having a mental health condition (Hashmi et al., 2024; Jokela et al., 2013), and in Australia, people residing in socially disadvantaged areas are more likely to have symptoms of anxiety and depression, are more likely to self-harm, and are more likely to die by suicide (Australian Institute of Health and Welfare, 2024; Hashmi et al., 2021; Staples et al., 2022). Digital psychology services, a particular subset of digital mental health services that provide evidence-based, therapist-supported services online or via the telephone, have the potential to overcome some of the barriers to care arising from low SES. However, no studies have directly examined the impact of SES on engagement with digital psychology services.

There is now a large body of evidence showing that psychological assessment and treatment provided via the internet or by telephone is effective, acceptable to patients, and can be successfully delivered to very large numbers of patients as part of routine care (Etzelmueller et al., 2020; Hadjistavropoulos et al., 2014; Hedman-Lagerlöf et al., 2023; Staples et al., 2022; Titov et al., 2020). The aim of the current study was to examine whether socioeconomic status affected engagement and clinical outcomes with a nationally delivered and government-funded digital psychology service. Residential postcodes were used to assign patients to a socioeconomic group based on the Index of Economic Resources published by the Australian Bureau of Statistics (2018). Engagement was measured as the proportion of patients starting and completing an online treatment course designed for symptoms of anxiety and depression (Dear et al., 2016; Dear et al., 2015). Treatment benefits were measured by examining outcomes on standardised symptom measures and overall patient satisfaction.

## 2 Methods

### 2.1 Participants and study design

This study is a secondary retrospective analysis of data collected at the MindSpot Clinic between 2013 and 2019. It includes all patients who completed an assessment and enrolled in an online, therapist-guided, treatment course for depression and anxiety between 2013 and 2019. At registration, patients provided informed consent for their deidentified data to be analysed. Patients were aged 18 years or older, Australian residents, and had self-reported symptoms of anxiety or depression. Approval to conduct the study was provided by the Macquarie University Human Research Ethics Committee (approval number 5201200912).

Residential postcodes were used to assign patients to a group based on tertiles of the Index of Economic Resources (IER), which was used as the proxy for socioeconomic disadvantage. The IER is one of four indexes produced by the Australian Bureau of Statistics (Australian Bureau of Statistics, 2018) to rank geographical areas in Australia by socioeconomic disadvantage. The indexes are highly correlated, and an a priori decision was made to explore the association between the IER and utilization of a digital psychology service. The IER summarises variables from the 2016 Australian Census related to income and housing, variables which are not directly surveyed in the MindSpot assessment, but are known to affect mental health (Hashmi et al., 2024).

### 2.2 Setting and procedure

The MindSpot Clinic is a digital psychology service funded by the Australian Government, and services are provided at no cost to patients. The clinical model is described in detail elsewhere (Titov et al., 2020; Titov et al., 2017; Titov et al., 2015). Briefly, patients completed an online assessment comprising standardised and non-standardised questionnaires related to demographics, symptoms and service use. They then enrolled in an online transdiagnostic course designed to treat symptoms of both anxiety and depression (Dear et al., 2016; Dear et al., 2015). All patients had access to therapist support at the time of assessment and throughout treatment. Courses consisted of five lessons delivered over eight weeks.

### 2.3 Measures of patient characteristics

The online assessment comprises a series of non-standardised self-report questionnaires designed to identify patient demographics, self-reported symptoms, and service use (Titov et al., 2020). The demographic variables included in the current study were age, gender, location, cultural background, employment, education, and relationship status. Other patient characteristic included in the current study were self-reported symptoms, risk, psychosocial stressors, treatment and service use history, and reason for accessing a digital psychology service.

### 2.4 Treatment outcome measures

Three standardised symptom scales were analysed in the current study: the Patient Health Questionnaire 9-item (PHQ-9), Generalised Anxiety Disorder 7-item (GAD-7) and the Kessler 10-item (*K*−10). The PHQ-9 measures symptoms of depression on a scale of 0 to 27, with scores ≥10 indicating a likely diagnosis of depression (Kroenke et al., 2001). The GAD-7 measures symptoms of generalised anxiety disorder and is sensitive to panic disorder and social phobia. Symptoms are measured on a scale of 0 to 21, and scores ≥8 indicate an anxiety disorder (Spitzer et al., 2006). The K-10 measures non-specific psychological distress on a scale of 10 to 50. Scores ≥22 suggest clinically relevant levels of distress (Australian Bureau of Statistics, 2003; Kessler et al., 2002).

The PHQ-9 and GAD-7 were administered at assessment, day one of the course and weekly throughout treatment. The K-10 was administered at assessment, day one, and mid-treatment. All questionnaires were administered at post-treatment (primary endpoint) and three months after treatment concluded (secondary endpoint). Satisfaction was also measured at post-treatment. The satisfaction questions reported in the current study are “Would you recommend this course to others?” and “Was it worth your time doing this course?”

### 2.5 Statistical analyses

Data were analysed using SPSS version 29. A significance level of 0.05 was used for all tests, with Bonferroni adjustments applied for multiple comparisons. Three groups were compared: Low SES (*n* = 7345), Mid SES (*n* = 7696), and High SES (*n* = 6,520). Group differences in patient characteristics at assessment were examined using chi-square analysis for categorical variables and ANOVA for continuous variables.

Treatment outcomes were examined for patients who started the lessons. Generalised Estimating Equation (GEE) models with Wald's χ2 as the test for significance were used to examine changes on the PHQ-9, GAD-7, and K-10, from assessment to post-treatment and assessment to 3-month follow-up. The GEE models assume data were missing at random, and baseline symptoms and lesson completion were included as covariates (Titov et al., 2020). An unstructured working correlation matrix and maximum likelihood estimation were used, and gamma distribution with a log link response scale was specified to address positive skewness in dependent variable distributions.

Cohen's d effect sizes were used to examine the clinical significance of improvements over time on the treatment measures. Effect sizes were estimated for all patients who started treatment using the estimated marginal means obtained from the GEE models. A measure of reliable deterioration was obtained for patients who completed post-treatment (Jacobson and Truax, 1991) and was defined as an increase in scores at post-treatment of at least one standard deviation (≥6 on the PHQ-9; ≥5 on the GAD-7, and ≥ 7 on the K-10).

## 3 Results

### 3.1 Sample distribution

**Fig. 1:**
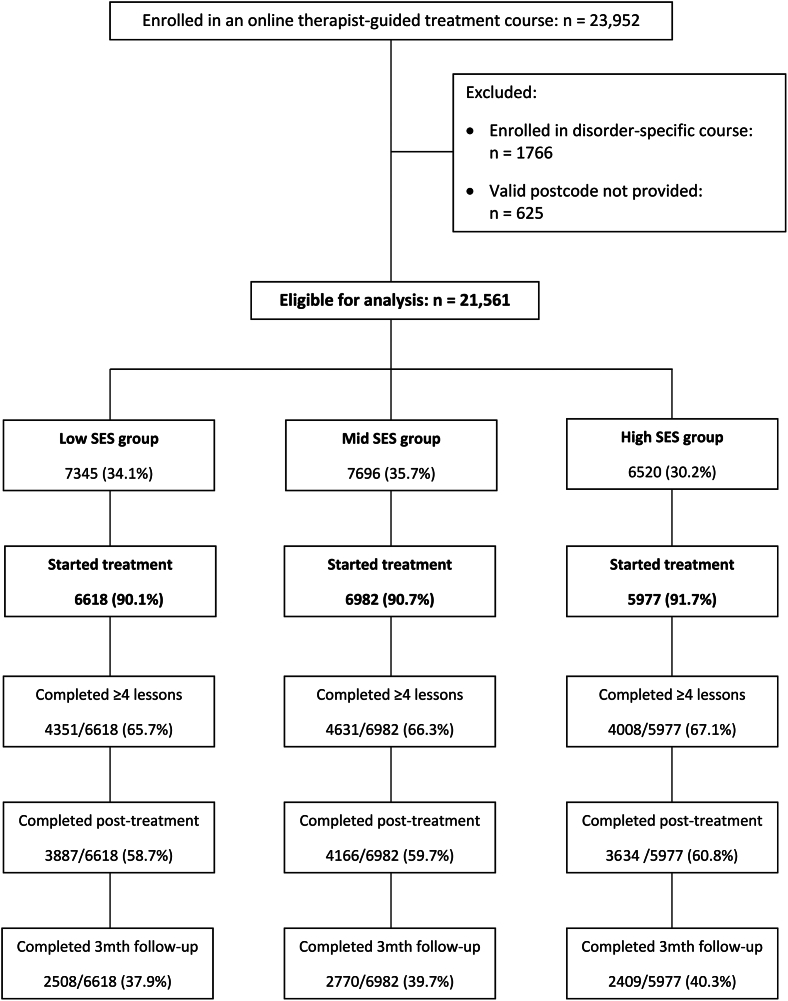
Patient flow. Low SES: lowest tertile on the Index of Economic Resources (IER); Mid SES: middle IER tertile; High SES: highest IER tertile. Course completion defined as 4 or more lessons. Post-treatment completion defined as completion of post-treatment symptom questionnaires.

Patients in the low and mid SES groups were over-represented in the sample of patients accessing treatment (Fig. 1). A total of 34.1 % of patients resided in the low SES group, and 35.7 % resided in the mid SES group. Both proportions are above the expected distribution of 33.3 % per tertile. The corresponding under-representation was observed in the high SES group, which comprised 30.2 % of the sample.

### 3.2 Demographic characteristics

Overall mean age of the sample was 39.5 (SD 13.7). There was a significant difference between mean ages of the SES groups (*F* = 27.1, *p* < .001), with the low SES group more likely to be younger (M 38.5, SD 13.6) compared to the mid SES and high SES groups (M 40.0, SD 13.8 for both). There were no differences across groups in gender distribution. As expected, groups differed in geographical distribution, as low SES areas are more likely to be regional or remote parts of Australia, and high SES areas are more likely to be major cities. There were also small but statistically significant differences between groups for cultural background, employment, education, and marital status. Patients in the low SES group were more likely to be younger and seeking employment, and less likely to be married. Patients in the high SES group were more likely to be born overseas, be married, and report a university qualification. They were less likely to report Indigenous status and less likely to be receiving the Disability Support Pension. Demographic characteristics are shown in Table 1.

**Table 1:** Demographic characteristics.

	Low SES		Mid SES		High SES		Significance
N	7345		7696		6520		
	n	%	n	%	n	%	
Gender							
Male	2123	28.9	2182	28.4	1934	29.7	ꭙ^2^ = 4.5, *p* = .343
Female	5193	70.7	5490	71.3	4568	70.1	
Other	29	0.4	224	0.3	18	0.3	

Location							
Major city	4275 ^a^	58.2	5358 ^b^	69.6	5648 ^c^	86.6	ꭙ^2^ = 1386.3, *p* < ·001
Regional	2819 ^a^	38.4	2146 ^b^	27.9	856 ^c^	13.1	
Remote	251 ^a^	3.4	192 ^b^	2.5	16 ^c^	0.2	

Cultural Background							
Born in Australia, non-Indigenous	5532 ^a^	75.3	5772 ^a^	75.0	4836 ^a^	74.2	ꭙ^2^ = 46.1, *p* < ·001
Aboriginal or Torres Strait Islander	144 ^a^	2.0	136 ^a^	1.8	53 ^b^	0.8	
Born overseas	1522 ^a^	20.7	1624 ^a^	21.1	1510 ^b^	23.2	
No answer	147	2.0	164	2.1	121	1.9	

Employment							
Paid employment	4291 ^a^	58.4	4741 ^b^	61.6	4076 ^b^	62.5	ꭙ^2^ = 96.7, *p* < ·001
Unemployed, seeking employment	855 ^a^	11.6	751 ^b^	9.8	598 ^b^	9.2	
Retired	408 ^a^	5.6	477 ^a^	6.2	405 ^a^	6.2	
Disability support pension	344 ^a^	4.7	300 ^a^	3.9	149 ^b^	2.3	
Other / no answer	1447 ^a^	19.7	1427	18.5	1292	19.8	

Education							
University degree	3276 ^a^	44.6	3450 ^a^	44.8	3169 ^b^	48.6	ꭙ^2^ = 41.3, *p* < ·001
Other tertiary qualification	2434 ^a^	33.1	2604 ^a^	33.8	2137 ^a^	32.8	
Secondary or below	1500 ^a^	20.4	1504 ^a^	19.5	1098 ^b^	16.8	
No answer	135	1.8	138	1.8	116	1.8	

Relationship Status							
Married (registered and de facto)	2967 ^a^	40.4	3655 ^b^	47.5	3385 ^c^	51.9	ꭙ^2^ = 222.8, *p* < ·001
Divorced	864 ^a^	11.8	1002 ^a^	13.0	679 ^b^	10.4	
Other / no answer	3514	47.8	3039	39.5	2456	37.7	

Significance column uses chi-square tests to compare SES groups on each categorical variable. Each superscript letter in the “n” columns denotes a subset of categories for that row variable whose proportions do not differ significantly from each other at the 0.05 level.

Table 2 shows differences in symptoms and service use. Groups showed small but statistically significant differences in self-reported baseline symptoms of depression and anxiety. The low SES group reported higher rates of depression and higher rates of suicidal thoughts. Interestingly, the highest rate of current anxiety was reported by the middle-ranking group. The low and mid SES groups reported more psychosocial stress associated with physical health and finances, and the high SES reported more vocational stress. There were also significant group differences in the main reasons for accessing a digital psychology service. Notably, a lack of local or affordable mental health services was the main response for the low SES group.

**Table 2:** Symptoms and service use reported at assessment.

	Low SES		Mid SES		High SES		Significance
N	7345		7696		6520		
	n	%	n	%	n	%	
Self-reported symptoms							
Current depression or low mood	5271 ^a^	71.8	5397 ^b^	70.1	4421 ^c^	67.8	ꭙ^2^ = 27.0, *p* < .001
Current anxiety or worry	6200 ^a^	84.4	6569 ^b^	85.4	5446 ^c^	83.5	ꭙ^2^ = 10.4, *p* < .05

Risk							
Suicidal thoughts	1810 ^a^	24.6	1814 ^a, b^	23.6	1468 ^c^	22.5	ꭙ^2^ = 11.1, *p* < .05
Current plan	92	1.3	75	1.0	65	1.0	ꭙ^2^ = 4.8, *p* = .310

Psychosocial stressors 1							
Vocational	4244 ^a^	57.8	4168 ^b^	54.2	3499 ^b^	53.7	ꭙ^2^ = 29.4, *p* < .001
Relationships	3866	52.6	4080	53.0	3335	51.2	ꭙ^2^ = 5.4, *p* = .069
Physical health	3014 ^a^	41.0	3160 ^a^	41.1	2449 ^b^	37.6	ꭙ^2^ = 23.0, *p* < .001
Finances	2433 ^a^	33.1	2441 ^a^	31.7	1921 ^b^	29.5	ꭙ^2^ = 21.7, *p* < .001
	
Treatment and service use history							
Current psychotropic medication	2445 ^a, b^	33.3	2682 ^b^	34.8	2073 ^a^	31.8	ꭙ^2^ = 16.4, *p* < .01
Never spoken to mental health professional	1710 ^a, b^	23.3	1691 ^b^	22.0	1598 ^a^	24.5	ꭙ^2^ = 14.0, *p* < .01


Main reason for accessing a digital psychology service 2							
n	5175		5430		4558		
Convenience, able to access immediately	1182 ^a^	22.8	1313 ^a, b^	24.2	1154 ^b^	25.3	ꭙ^2^ = 8.2, *p* < .05
Preference for privacy and anonymity	1104	21.3	1160	21.4	1023	22.4	ꭙ^2^ = 2.3, *p* = .324
Lack of local or affordable mental health services	1194 ^a^	23.1	1166 ^a^	21.5	800 ^b^	17.6	ꭙ^2^ = 46.8, *p* < .00

Significance column uses chi-square tests to compare SES groups on each categorical variable.

1 Multiple options can be selected.

2 Question introduced in 2015. Each superscript letter in the “n” columns denotes a subset of categories for that row variable whose proportions do not differ significantly from each other at the 0.05 level.

### 3.3 Treatment engagement and outcomes

Patients in the highest SES areas were slightly more likely to start the online course (ꭙ^2^ = 10.3, *p* = .006). However, once patients started treatment, there were no group differences in lesson completion (ꭙ^2^ = 2.4, *p* = .297), or questionnaire completion at post-treatment ((ꭙ^2^ = 5.6, *p* = .062). There was a very small but statistically significant difference in engagement again at 3-month follow-up (ꭙ^2^ = 8.4, *p* = .015), with the rate of completion for the follow-up questionnaires slightly higher for the high SES group.

**Fig. 2:**
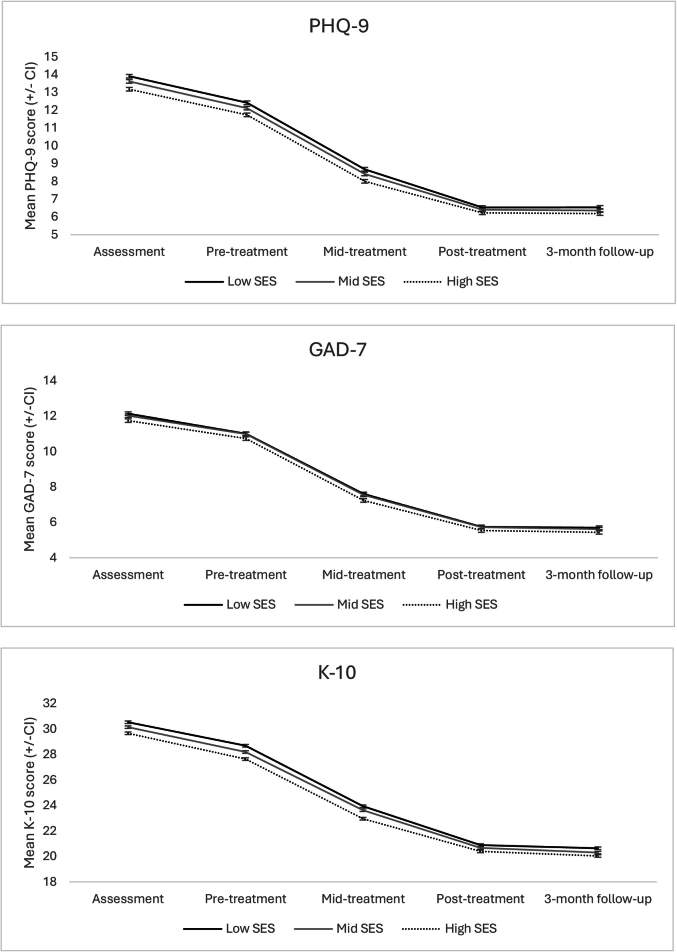
Estimated marginal means over time, for patients grouped by socioeconomic status.

Fig. 2 shows group means over time on the PHQ-9, GAD-7 and K-10. Mean scores for all groups were below clinical cut-offs by post-treatment on all measures, and remained below cut-offs at three-month follow-up. Effect sizes are shown in Table 3. For all groups and for all measures, large effect sizes were observed at post-treatment (between 1.36 and 1.47) and at three-month follow-up (between 1.34 and 1.51). Reliable deterioration rates were low (1.3 % to 1.6 % for the PHQ-9; 1.9 % to 2.1 % on the GAD-7; and 1.6 % to 2.4 % on the K-10).

**Table 3:** Means and clinical significance.

		Estimated Marginal Means			Effect sizes from assessment		Reliable deterioration
	N	Assessment	Post-treatment	3-month follow-up	to post-treatment	to 3-month follow-up	
PHQ-9							
Low SES	6618	13.9 (5.8)	6.5 (4.1)	6.5 (4.4)	1.47 1.43–1.51	1.44 1.40–1.48	1.6 % (61/3887)
Mid SES	6982	13.6 (5.9)	6.4 (4.1)	6.4 (4.2)	1.42 1.38–1.45	1.41 1.37–1.44	1.3 % (52/4166)
High SES	5977	13.1 (5.9)	6.2 (4.1)	6.2 (4.3)	1.36 1.32–1.40	1.34 1.30–1.38	1.3 % (47/3634)

GAD-7							
Low SES	6618	12.1 (4.9)	5.7 (3.6)	5.7 (3.8)	1.49 1.45–1.53	1.46 1.42–1.50	2.1 % (81/3887)
Mid SES	6982	12.0 (5.0)	5.7 (3.6)	5.6 (3.7)	1.45 1.41–1.48	1.46 1.42–1.49	1.9 % (77/4166)
High SES	5977	11.7 (5.0)	5.5 (3.5)	5.4 (3.6)	1.44 1.40–1.48	1.45 1.41–1.49	2.1 % (75/3634)

K-10							
Low SES	6618	30.5 (6.9)	20.9 (6.1)	20.6 (6.5)	1.47 1.44–1.51	1.48 1.44–1.52	2.0 % (76/3887)
Mid SES	6982	30.1 (6.8)	20.7 (6.0)	20.3 (6.2)	1.47 1.43–1.50	1.51 1.47–1.54	2.4 % (99/4166)
High SES	5977	29.7 (6.8)	20.4 (5.9)	20.0 (6.2)	1.46 1.42–1.50	1.49 1.45–1.53	1.6 % (59/3634)

Satisfaction rates were also high for all groups. Of the patients who completed the satisfaction questionnaires, 97 % of each group would recommend MindSpot to a friend (low SES group: 3799/3929; mid SES group: 4115/4263; high SES group: 3569/3684). The proportion reporting that the treatment was worth their time was also high, 96 % of the mid SES group (4106/4258) and 97 % of the low (3792/3919) and high groups (3559/3675).

## 4 Discussion

This study examined whether socioeconomic status was associated with differences in demographic characteristics and treatment outcomes, for patients accessing a national digital psychology service as part of routine care. For the study, a large sample of patients (*n* = 21,561) were categorised into three groups based on a government-maintained geographic index of economic resource characteristics. The low and mid SES groups comprised 34.1 % and 35.7 % of the sample respectively. Patients in the low SES group were more likely to be younger, unemployed, and live in regional or remote Australia. Previous studies show these variables can be associated with worse mental health (Australian Institute of Health and Welfare, 2024; Hashmi et al., 2024; Mihalopoulos et al., 2021; Staples et al., 2022). Consistent with this, 71.8 % of the low SES group reported symptoms of depression or low mood at assessment, compared to 67.8 % of the high SES group.

There were also significant group differences in the reasons given for choosing an online service. Patients in the low and mid SES groups reported a lack of local or affordable mental health services as the main reason for accessing a digital psychology service. These patients were also more likely to report psychosocial stress due to physical health, work, or finances. Taken together, these findings confirm some of the barriers to mental health care faced by socioeconomically disadvantaged groups (Amos et al., 2023; Australian Institute of Health and Welfare, 2024), and the importance of a health system that can provide equitable services that are easily accessible at no cost to the consumer. In addition to optimising access to mental health services, the impact of psychosocial stressors in the low and mid SES groups points to the importance of being able to integrate mental health services with other support providers, such as those related to housing, employment, or education (Titov et al., 2025).

Despite some differences at baseline, all groups responded well to treatment, with average symptom scores reducing to below clinical cut-offs on measures of anxiety and depression, and symptom improvements were maintained to three month follow up. Clinical deterioration rates were low, and satisfaction rates were high across groups. Digital psychology services can be effectively delivered as part of routine care to many groups that may otherwise face disadvantage, including Indigenous groups (Titov et al., 2019), migrants (Kayrouz et al., 2020), and rural or remote patients (Staples et al., 2025). Similarly, this study demonstrates that excellent clinical outcomes can be achieved regardless of socioeconomic status.

The main strength of the study was the comprehensive measurement of symptoms in a large sample of patients accessing treatment as part of routine care, and the use of a government-maintained index to define SES status (Australian Bureau of Statistics, 2018). At the same time, it is important to note that the index summarises characteristics at an area level rather than an individual level. There can be great individual diversity within a geographical area, and this means that the outcomes do not directly reflect the relative advantage or disadvantage of any individual (Australian Bureau of Statistics, 2018).

We also note that other factors related to socioeconomic status may affect how individuals interpret symptom or satisfaction measures. For example, socioeconomic disparities can be associated with lower levels of health literacy (Stormacq et al., 2018), although recent research encouragingly showed that in a sample of treatment-seeking adults accessing a digital psychology service, health literacy difficulties were not associated with treatment uptake or symptom improvements (Fisher et al., 2025). Further studies might include more direct, individual-level identification of SES, for example by combining education, income and housing data provided by participants. The pre-COVID timeframe of the study also limits the generalisability of the results, and it would be of interest to re-run the study to include registrants during the COVID-19 pandemic and its aftermath.

Despite these limitations, this is the first study to explore the relationship between access to economic resources and mental health, in such a large sample of people accessing a digital psychology service as part of routine care. There are clear policy implications for this research: appropriately governed digital psychology services are able to provide effective, evidence-based treatment across socioeconomic levels. Their relative low cost and scalable nature (Titov et al., 2025) means that they can provide mental health care to people who otherwise report a lack of local or affordable options.

## 5 Conclusion

Socioeconomic disadvantage is often associated with higher rates of mental disorder, psychosocial difficulties, and barriers restricting access to treatment. Our findings indicate that digital psychology services can provide effective and acceptable support across socioeconomic levels. While further research is needed, these results support suggestions that scalable digital psychological services have the potential to improve the equity of access to mental health care.

## Declaration of competing interest

All authors are involved in developing and delivering digital mental health services. The authors have no other disclosures.
